# HCC recurrence in liver transplants treated with hypothermic oxygenated machine perfusion: An international matched cohort study

**DOI:** 10.1016/j.jhepr.2026.101732

**Published:** 2026-01-21

**Authors:** Janina Eden, Philip C. Müller, Christoph Kuemmerli, Marco Bongini, Francesca Albanesi, Carlo Sposito, Gabriela Berlakovich, Bettina M. Buchholz, Florin Botea, Stefania Camagni, Matteo Cescon, Umberto Cillo, Fabio Colli, Philippe Compagnon, Luciano G. De Carlis, Riccardo De Carlis, Fabrizio Di Benedetto, Jule Dingfelder, Dulce Diogo, Daniele Dondossola, Moritz Drefs, Jiri Fronek, Giuliana Germinario, Enrico Gringeri, Christiano Guidetti, Georg Györi, Matej Kocik, Efrayim H. Küçükerbil, Dionysios Koliogiannis, Hwai-Ding Lam, Georg Lurje, Paolo Magistri, Diethard Monbaliu, Mostafa el Moumni, Beat P. Müller, Damiano Patrono, Wojciech G. Polak, Robert J. Porte, Matteo Ravaioli, Michel Rayar, Renato Romagnoli, Gustaf Sörensen, Deniz Uluk, Pierre A. Clavien, Vincenzo Mazzaferro, Philipp Dutkowski, Vincent E. de Meijer

**Affiliations:** 1 Department of Surgery, Section of HPB Surgery and Liver Transplantation, University of Groningen and University Medical Center Groningen, Groningen, the Netherlands; 2 UMCG Comprehensive Transplant Center, Groningen, the Netherlands; 3 Department of Visceral Surgery, University of Basel, Clarunis, Basel, Switzerland; 4 Division of Transplantation, Medical University of Vienna, Vienna, Austria; 5 Department of Visceral Transplantation, University Medical Center Hamburg-Eppendorf, Hamburg, Germany; 6 Fundeni Clinical Institute, Center of General Surgery and Liver Transplantation; "Titu Maiorescu" University, Bucharest, Romania; 7 Department of Organ Failure and Transplantation, ASST Papa Giovanni XXIII, Bergamo, Italy; 8 Department of General Surgery and Transplantation, IRCCS Azienda Ospedaliero-Univeritaria of Bologna, University of Bologna, Bologna, Italy; 9 Chirurgia Generale 2, Hepato-Biliary-Pancreatic Unit and Liver Transplant Center, Padova University Hospital, Padova, Italy; 10 General Surgery 2U, Liver Transplant Centre, Azienda Ospedaliero Universitaria Città della Salute e della Scienza di Torino, Turin, Italy; 11 Department of Transplant Surgery, University of Geneva, Geneva, Switzerland; 12 Department of General Surgery and Transplantation, ASST Grande Ospedale Metropolitano Niguarda, Milan, Italy; 13 Hepato-Pancreato-Biliary Surgery and Liver Transplantation Unit, University of Modena and Reggio Emilia, Modena, Italy; 14 Adult Liver Transplantation Unit, Department of Surgery and Gastroenterology, Coimbra Hospital and University Center, Coimbra, Portugal; 15 General and Liver Transplant Surgery Unit, Fondazione IRCCS Ca’ Granda Ospedale Maggiore Policlinico, and Department of Pathophysiology and Transplantation Università degli Studi di Milano, Milan, Italy; 16 Department of Transplant Surgery, University of Munich Grosshaderm, Germany; 17 Transplant Surgery Department, Institute for Clinical and Experimental Medicine (IKEM), Prague, Czech Republic; 18 Erasmus MC Transplant Institute, University Medical Center Rotterdam, Division of HPB and Transplant Surgery, Rotterdam, the Netherlands; 19 Department of Surgery, Leiden University Medical Center, Leiden, the Netherlands; 20 Department of Surgery, Universitätsklinikum Heidelberg, Heidelberg, Germany; 21 Department of Abdominal Transplantation, Leuven Transplant Center, University Hospitals Leuven, Leuven, Belgium; 22 Department of Surgery, Section of Epidemiology and Statistics, University of Groningen and University Medical Center Groningen, Groningen, the Netherlands; 23 CHU Rennes, Service de Chirurgie Hépatobiliaire et Digestive, Rennes, France; 24 Transplant Institute, Sahlgrenska University Hospital, Gothenburg, Sweden; 25 Swiss HPB and Transplant Center, Department of Visceral Surgery and Transplantation, University Hospital Zurich, Zurich, Switzerland; 26 Instituto Nazionale Tumori IRCCS Milano, University of Milan, Italy

**Keywords:** Hepatocellular carcinoma, HCC recurrence, liver transplantation, hypothermic oxygenated machine perfusion, HOPE, ischemia reperfusion injury

## Abstract

**Background & Aims:**

Liver transplantation (LT) for hepatocellular carcinoma (HCC) is performed worldwide, with 5-year survival rates of approximately 70%. However, post-transplant HCC recurrence occurs in 15-20% of recipients. We aimed to evaluate, for the first time, long-term recurrence-free survival in a large international cohort of patients undergoing LT for HCC using grafts treated with hypothermic oxygenated machine perfusion (HOPE).

**Methods:**

This observational *post hoc* analysis of the multicenter European HOPE-REAL study (NCT05520320) included adult recipients with HCC (N = 599) who received a liver from either a donation after brain death (DBD) or donation after circulatory death (DCD) donor, preserved using HOPE, dual-HOPE (DHOPE), or normothermic regional perfusion followed by HOPE (NRP-HOPE) between 2012 and 2022. Propensity score matching was used to compare outcomes between HCC and non-HCC recipients within the HOPE-REAL cohort, and between HOPE-treated HCC recipients and an external control cohort receiving non-perfused livers (n = 484).

**Results:**

The overall HCC recurrence rate in the HOPE-REAL cohort was 6.9% (41/599), with no significant difference between DBD and DCD liver transplants (7.1% [25/350] *vs.* 6.4% [16/249]; *p =* 0.346). One-, 3-, and 5-year overall survival rates were 92%, 86%, and 81%, while recurrence-free survival rates were 90%, 83%, and 78%, respectively. Five-year overall survival was similar between 347 HOPE-treated HCC recipients (82%) and 347 matched non-HCC recipients (84%) (*p =* 0.625). In contrast, compared to an external cohort of 312 non-perfused HCC recipients, 5-year overall survival was significantly higher in 312 matched HOPE-treated HCC recipients (74% *vs.* 84%; *p =* 0.034).

**Conclusions:**

HCC recurrence was rare after transplantation of livers treated with HOPE. Long-term survival in HOPE-treated HCC recipients was significantly better than in those receiving non-perfused livers, and comparable to outcomes in non-HCC recipients. These findings warrant validation in a randomized clinical trial.

**Impact and implications:**

This *post hoc* analysis of the HOPE REAL study demonstrates, for the first time, low hepatocellular carcinoma (HCC) recurrence rates in a large cohort of hypothermic oxygenated machine perfusion-treated liver transplant recipients with HCC, and significantly better survival outcomes compared to matched recipients of non-perfused grafts. These findings may have important implications, particularly as tumor-related indications for liver transplantation continue to rise. Machine liver perfusion could emerge as a novel strategy to improve oncological outcomes in high-risk cancer conditions after transplantation, potentially via mitigation of inflammation and reduced tumor cell seeding.

**Clinical trial number:**

NCT05520320

## Highlights

• HOPE treatment was identified as an independent significant protective factor against HCC recurrence.
• Among patients with HCC, 5-year suvival and recurrence -free survival was higher in matched recipients of HOPE treated DBD grafts than non-perfused DBD grafts (84% vs. 74% and 77 vs. 67%, respectively).
• In contrast, survival was not different between HOPE treated HCC recipients and matched non HCC recipients ( 82% vs 84% at 5 years).

## Graphical abstract

**Figure:**
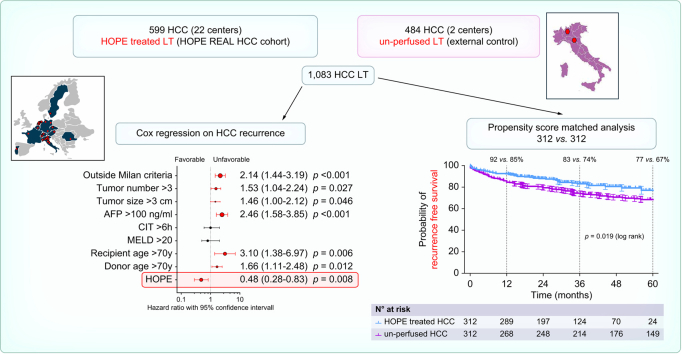

## Introduction

Hepatocellular carcinoma (HCC) is the most common primary liver cancer and the third leading cause of cancer-related mortality worldwide. Liver transplantation (LT) is the treatment of choice for early-stage HCC, offering excellent outcomes, with 5-year survival rates exceeding 70%.^1^ However, despite the implementation of strict selection criteria, post-LT HCC recurrence occurs in up to 15-20% of recipients and is associated with poor outcome.^2^ HCC recurrence typically arises within the first 2 years after LT, frequently in the transplanted liver but also at other locations such as the lungs and bones.^3^ The mechanisms driving HCC recurrence after LT remain incompletely understood. Ischemia-reperfusion injury (IRI) has been identified as a contributing factor, alongside immunosuppressive therapy and tumor-related parameters, such as tumor size, tumor number, vascular infiltration, and serum alpha-fetoprotein (AFP) levels.^4^ The current rationale is that IRI promotes tumor seeding and tumor growth by creating an inflammatory environment, triggered through the release of danger signals, adhesion molecules, and by exacerbation of hypoxic conditions.^5^^,^^6^ *Ex situ* machine perfusion approaches, such as hypothermic oxygenated machine perfusion (HOPE), represent a novel and straightforward strategy to reduce graft inflammation prior to transplantation,^7^ and have therefore recently been suggested as a potential intervention to mitigate cancer recurrence due to their anti-inflammatory effects.^8^ We hypothesized that HOPE-treated livers would demonstrate a lower risk of HCC recurrence compared to non-perfused livers. Here, we report for the first time the impact of HOPE treatment on HCC recurrence in a large, international cohort of livers donated after brain death (DBD) and circulatory death (DCD).

## Patients and methods

### Study design

This observational cohort study is a *post hoc* analysis of the multicenter European HOPE-REAL study (NCT05520320).^9^ It includes adult recipients with HCC from 22 European LT centers who received either a DBD or DCD liver graft treated with HOPE, dual-HOPE (DHOPE), or normothermic regional perfusion followed by HOPE (NRP-HOPE) between 2012 and 2022. Eligibility criteria for participating centers have been detailed previously;^9^ in summary, centers were eligible after performing at least 20 HOPE procedures, and all consecutive HOPE cases outside clinical trials were included in this analysis. Data collection extended until December 31, 2022, ensuring a minimum follow-up time of 12 months for each patient.

The primary endpoint of this study was recurrence-free survival after LT, defined as survival without evidence of HCC recurrence. Time-to-event data were measured starting from the date of transplantation, and patients were censored at the end of the observation period when alive without evidence of recurrence. HCC recurrence was defined as tumor recurrence in the transplanted liver or disseminated disease (metastases at other sites), as detected through cross-sectional imaging or ultrasonography. Histological confirmation was obtained in the cases with ambiguous findings. Secondary endpoints included patient survival following LT. We collected donor, graft and recipient parameters, including donor age, total donor warm ischemia time (interval between donor withdrawal of life support and cold flush), functional warm ischemia time (interval between blood pressure <50 mmHg and the start of cold *in situ* flush), cold storage preservation time, recipient age, and recipient laboratory model for end-stage liver disease (MELD) score.

The most recent AFP value prior to transplantation was recorded. Tumor number, tumor size (diameter of the largest lesion), and vascular invasion were assessed on the pathological specimen to account for potentially undetected tumors on pre-transplant imaging. To estimate oncological risk profiles, we calculated risk scores for each patient, including risk assessments based on the balance of risk score, donor risk index, and the UK DCD risk score, and tumor stage assessments based on Milan criteria (within *vs*. outside)^10^ and the AFP model (score ≤2 *vs*. >2).^11^

Data from the HCC cohort were first compared to non-HCC patients within the HOPE-REAL cohort. In a second analysis, survival outcomes of HOPE-treated HCC DBD liver transplants were compared to those of an external cohort of patients with HCC who received non-perfused DBD livers. This external cohort included patients transplanted within the last 5 years at two Italian centers: the Instituto Nazionale dei Tumori IRCCS, University of Milan, and the Hepato-Pancreato-Biliary Surgery and Liver Transplantation Unit, University of Modena. Both comparisons were performed using propensity score matching.

### Statistical analysis

Propensity score analysis was performed using R software (version 4.4.3). For the HCC *vs.* non-HCC comparison within the HOPE-REAL cohort, 1:1 matching was conducted based on the following potential confounders: HOPE type (DHOPE, HOPE, NRP-HOPE), graft type (DBD, DCD), graft risk category (benchmark DBD, standard DBD, extended criteria DBD, low-risk DCD, high-risk DCD, futile DCD), donor age, recipient age, recipient MELD, re-transplant status (yes, no), and cold ischemia time. For the comparison between HOPE-treated and non-perfused patients with HCC, 1:1 matching was performed with the confounders AFP, tumor size, tumor number, recipient age, recipient MELD, donor age and cold ischemia time. For both comparisons, propensity scores were generated using the specified variables for each patient using a multivariate regression model. We used the nearest neighbor method with a caliper width of 0.1 of the standard deviation of the logit of the estimated propensity score. The standardized mean difference was less than 0.25 in both comparisons, indicating balance between the cohorts.

Numeric variables were compared using the Mann-Whitney *U* test, while categorical variables were analyzed using Fisher’s exact test. Distribution was assessed visually and using a Shapiro–Wilk test. Recurrence-free survival and patient survival were calculated using Kaplan-Meier analysis and compared using the log-rank test. Cox regression analysis was performed to adjust for the abovementioned matching variables of prognostic relevance. The proportional hazard assumption was assessed using Schoenfeld residuals, which were inspected visually (Schoenfeld residual plots) and tested for a non-zero slope.

## Results

**Fig. 1:**
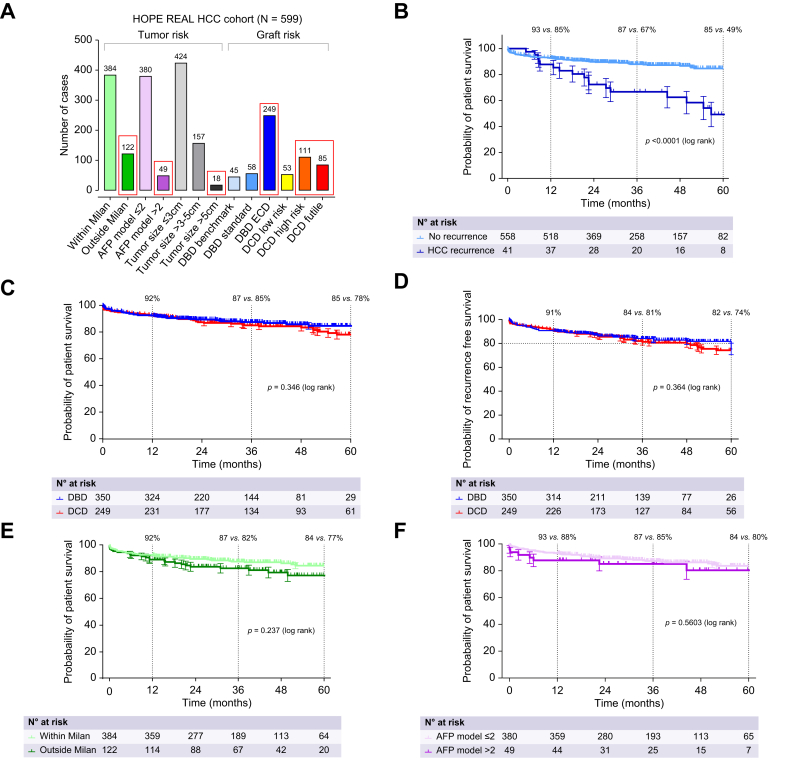
Risk profile and survival outcomes of HCC recipients in the HOPE-REAL study. (A) Risk profile of the HCC cohort in the HOPE-REAL study. Tumor risk is reported by the number of cases within/outside Milan, AFP model ≤2 or >2, tumor size ≤3 cm, 3-5 cm, or >5 cm. The graft risk is shown by the number of cases receiving low-risk, high-risk or futile DCD livers according to the UK DCD score (red circles visualize higher risk). (B) Patient survival of the HCC cohort with and without HCC recurrence. (C,D) Separate survival analysis (patient and recurrence-free survival) for DCD and DBD livers. (E,F) Patient survival rates within advanced tumor stages, *i.e.* outside Milan criteria and AFP model ≥2. AFP, alpha-fetoprotein; DBD, donation after brain death; DCD, donation after circulatory death; HCC, hepatocellular carcinoma.

This *post hoc* analysis from the HOPE-REAL study reports on 599 patients (49.8%) who underwent LT for HCC and on 603 LT recipients (50.2%) without HCC.^9^ The median tumor size in the HCC cohort was 2.0 cm (IQR 1.0-3.0 cm), with a median tumor number of 2 (IQR 1-3), and a median AFP level of 6 ng/ml (IQR 3.2-29.6 ng/ml) (Fig. 1A). Two thirds of these cases (64%) fell within the Milan criteria, corresponding to 380/599 patients (63%) with a low-risk AFP model score (≤2). The median donor age was 63 years (IQR 52-74 years), and the median recipient age was 61 years (IQR 55-66 years). DBD LT was performed in 350 patients (58%), and 249 (42%) recipients received DCD livers (231/249 [93%] Maastricht type III; 18/249 [7%] Maastricht V), including 196 of 599 livers (33%) ranked in the high-risk or futile category according to the UK DCD risk score. Most livers underwent HOPE treatment (46%), followed by DHOPE (39%) and NRP-HOPE (15%). Eighty percent of livers were perfused with Liver Assist® (XVIVO), 18% were perfused with Vitasmart® (Bridge to Life). The HOPE/DHOPE procedure was performed for a median of 140 min (106-196 min) with a median perfusion flow rate of 240 ml/min (150-250 ml/min) and a perfusate oxygenation degree of 80 kPa. Additional descriptive parameters, stratified by DBD and DCD cases, are summarized in Table 1.

**Table 1:** Descriptive parameters of the HCC cohort in the HOPE REAL study.

Parameter	Overall (N = 599)	DBD (n = 350)	DCD (n = 249)	*p* value∗
Donor age (years)	63 (52-74)	69 (55-78)	57 (48-66)	<0.001^1^
Donor BMI	26 (24-29)	26 (24-29)	26 (24-28)	0.157^1^
Tumor size (cm)	2 (1-3)	1.8 (1-2.7)	2 (1.2-3)	<0.001^1^
Tumor number	2 (1-3)	2 (1-3)	2 (1-3)	0.421
AFP (ng/ml)	6 (3.2-15.3)	5.3 (3-14.6)	6.2 (3.6-16.4)	0.079^1^
AFP model				
≤2 (%)	380/599 (63.4)	218/350 (62.3)	162/249 (65.1)	0.762^2^
>2 (%)	49/599 (8.2)	27/350 (7.7)	22/249 (8.8)	
Missing	170 (28.4)	105/350 (30)	65/249 (26.1)	
Milan criteria				
Within (%)	384/599 (64.1)	228/350 (65.1)	156/249 (62.7)	0.752^2^
Above (%)	122/599 (20.4)	70/350 (20)	52/249 (20.9)	
Missing	93/599 (15.5)	52/350 (14.8)	41/249 (16.4)	
Donor risk index	2.14 (1.89-2.52)	2.00 (1.77-2.19)	2.47 (2.05-2.77)	<0.001^1^
Functional warm ischemia time (min)	31 (25-40)	-	31 (25-40)	-
Cold ischemia time (min)	360 (264-471)	410 (294-502)	316 (241-406)	<0.001^1^
HOPE-type:				
HOPE (%)	273/599 (45.6)	162/350 (46.3)	111/249 (44.6)	<0.001^2^
DHOPE (%)	235/599 (39.2)	188/350 (53.7)	47/249 (18.9)	
NRP HOPE (%)	91/599 (15.2)	-	91/249 (36.5)	
Hope-time (min)	140 (106-196)	150 (116-200)	134 (100-192)	0.044^1^
Recipient age (years)	61 (56-66)	61 (55-66)	61 (57-67)	0.385
Recipient MELD	11 (8-15)	11 (9-16)	10 (8-13)	<0.001^1^
BAR	4 (3-5)	5 (3-7)	4 (3-5)	<0.001^1^
UK DCD	8 (5-11)	-	8 (5-11)	-
Peak AST (U/L)	805 (406-1,995)	679 (316-1,438)	1,120 (556-2,921)	<0.001^1^
Peak ALT (U/L)	598 (297-1,185)	454 (239-895)	846 (483-1,643)	<0.001^1^
Immunosuppression:				
mTor inhibitors (%)	12/599 (2%)	7/350 (2%)	5/249 (2%)	1.0
ICU stay (days)	4 (2-6)	5 (3-7)	3 (2-5)	<0.001^1^
Hospital stay (days)	15 (11-21)	15 (11-22)	15 (10-21)	0.152^1^
HCC recurrence (%)	41/599 (6.9)	25/350 (7.1)	16/249 (6.4)	0.732
Recurrence site§:				
Liver (n/%)	22/41 (54%)	14/25 (56%)	8/16 (50%)	0.856^3^
Lung (n/%)	5/41 (12%)	2/25 (8%)	3/16 (19%)	
Bones (n/%)	7/41 (17%)	5/25 (20%)	2/16 (13%)	
Other (n/%)	14/41 (34%)	6/25 (24%)	8/16 (50%)	

AFP, alpha-fetoprotein; ALT, alanine aminotransferase; AST, aspartate aminotransferase; BAR, balance of risk; DBD, donation after brain death; DCD, donation after circulatory death; DHOPE, dual hypothermic oxygenated machine perfusion; HCC, hepatocellular carcinoma; HOPE, hypothermic oxygenated machine perfusion; ICU, intensive care unit; MELD, model for end-stage liver disease; NRP, normothermic regional perfusion.

^4^Log-rank test.

∗ DCD *vs*. DBD.

§ Multiple counts.

1 Mann-Whitney *U* test.

2 Fisher exact test.

3 Chi-square test.

After a median follow-up time of 2.4 years, the overall HCC recurrence rate was 6.9%, with no significant difference between DBD (7.1%) and DCD (6.4%) liver transplants (*p =* 0.732; Table 1). Correspondingly, the 1-, 3-, and 5-year overall survival rates for the HCC cohort were 92%, 86%, and 81%, while recurrence-free survival rates were 90%, 83%, and 78%, respectively. Recipients with HCC recurrence had a significantly worse outcome (log-rank *p <*0.0001; Fig. 1B). Overall survival (log-rank *p =* 0.346; Fig. 1C) and recurrence-free survival (log rank *p =* 0.364; Fig. 1D) were not significantly different between DBD and DCD recipients. Likewise, post-transplant overall survival in HOPE-treated patients was not affected by more advanced tumor stages, *i.e*. overall survival was similar in HOPE-treated patients within *vs*. outside the Milan criteria (log rank *p =* 0.237; Fig. 1E), as well as in those with an AFP score up to 2 compared to those with a score exceeding 2 (log rank *p =* 0.560; Fig. 1F).

**Fig. 2:**
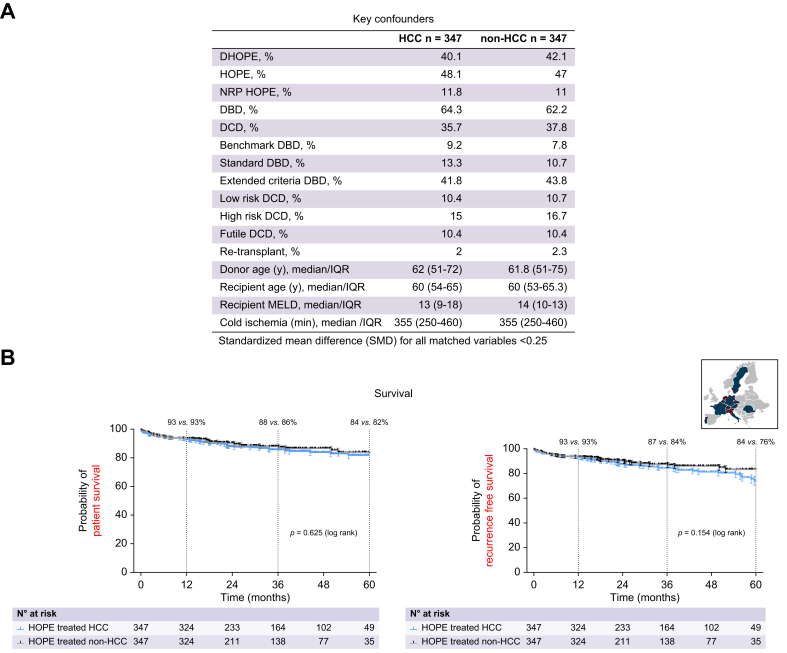
Propensity score-matched comparison of survival in HOPE-treated HCC *vs.* non-HCC recipients. (A,B) Adjusting for key confounders by propensity score matching in the HOPE-REAL cohort resulted in similar long-term overall and recurrence-free survival after liver transplantation for HOPE-treated HCC and non-HCC liver transplant recipients. DBD, donation after brain death; DCD, donation after circulatory death; DHOPE, dual hypothermic oxygenated machine perfusion; HCC, hepatocellular carcinoma; HOPE, hypothermic oxygenated machine perfusion; ICU, intensive care unit; MELD, model for end-stage liver disease; NRP, normothermic regional perfusion.

To assess the impact of HCC as the underlying indication for LT on long-term overall survival, we performed propensity score matching with adjustment for key confounders (Fig. 2A). Five-year overall survival and recurrence-free survival were comparable between 347 HOPE-treated HCC recipients (82%) and 347 matched non-HCC recipients (84%) (log rank *p =* 0.625 and *p =* 0.154; Fig. 2B).

**Fig. 3:**
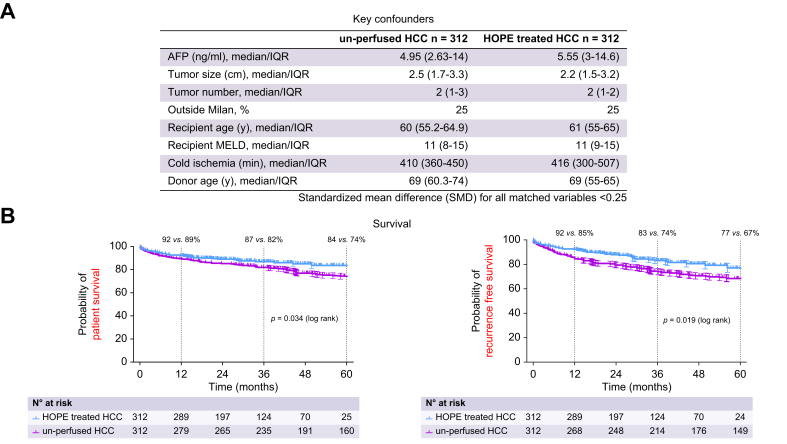
Propensity score-matched comparison of survival in HOPE-treated *vs.* non-perfused DBD HCC recipients. (A,B) Adjusting for key confounders by propensity score matching in the DBD subgroup of the HOPE-REAL HCC cohort and an external DBD non-perfused control group demonstrated significantly better long-term overall and recurrence-free survival after liver transplantation for HCC with HOPE-treated *vs.* non-perfused livers. AFP, alpha-fetoprotein; DBD, donation after brain death; DCD, donation after circulatory death; HCC, hepatocellular carcinoma; HOPE, hypothermic oxygenated machine perfusion.

A separate propensity score-matched analysis was conducted in a DBD subgroup to assess the impact of HOPE treatment in 312 patients with HCC from the HOPE-REAL cohort, compared to a contemporary external cohort of 312 patients with HCC who received non-perfused grafts (Fig. 3A). Five-year overall survival and recurrence free survival rates were significantly higher in the HOPE-treated DBD group compared to the non-perfused DBD control group (84% *vs.* 74% & 77 *vs.* 68% respectively; log rank *p =* 0.034 & *p =* 0.019; Fig. 3B), despite comparable tumor burden (Table 2). Accordingly, the overall HCC recurrence rate was 23/312 (7.4%) in the HOPE cohort and 59/312 (18.9%) in the non-perfused cohort (*p <*0.0001). Importantly, tumor recurrence in the transplanted liver graft (as the first site of recurrence) occurred in 35% (8/23) of HOPE-treated recipients compared with 59% (35/59) of non-perfused controls (*p =* 0.05).

**Table 2:** Tumor and transplant parameters in HCC liver transplant cohorts.

	HOPE REAL cohort 2012-2022	SRTR^12^ 2003-2020	ELTR^13^ 2006-2016	Cleveland^12^ 2000-2020	External cohort§ 2019-2024
Number of patients	599	26,232	6,720	547	484
Recipient MELD	11 (8-15)	12 (9-16)	13.1 (6.1)∗	12 (8-19)	10 (8-14)
Recipient age (years)	61 (56-66)	50 (55-65)	56.8 (8.6)∗	60 (56-65.5)	59 (54-64)
Outside Milan criteria (%)	122 (20.4)	1,820 (6.9)	2,894 (40.1)	100 (18.2)	162 (33.5)
Outside up to seven criteria (%)	78 (13)	992 (3.8)	1,717 (24.3)	42 (7.7)	92 (19.0)
>3 nodules (%)	84 (14)	-	1,540 (20.7)	-	84 (17.4)
Max size of noules >5 cm	18 (3)	-	760 (10.7)	-	67 (13.8)
Median tumor number	2 (1-3)	1 (1.2)	-	1 (1-2)	2 (1-3)
Median tumor size (cm)	2 (1-3)	2.5 (1.5-3.5)	-	2.3 (1.7-2.3)	2.1 (1.5-3.2)
Median AFP (ng/ml)	6 (3-15)	8 (4-26)	-	9.1 (4.6-29.6)	5.3 (3.0-14.6)
Median donor age (years)	63 (52-74)	44 (28-56)	-	43 (29-55)	66 (60-73)
Median donor BMI	26 (24-29)	37 (23-31)	-	27 (24-32)	-
DCD (%)	249 (41.6)	1,906 (7.2)	-	58 (10.8)	0
Median cold ischemia (h)	6 (4.4-7.9)	6 (4.8-7.9)	-	6.8 (5.8-8)	6.8 (6-7.3)

DCD, donation after circulatory death; ELTR, European Liver Transplant Registry; MELD, model for end-stage liver disease; SRTR, Scientific Registry of Transplant Recipients.

∗ Mean (SD).

§ Instituto Nazionale Tumori IRCCS, University of Milan, Hepato-Pancreato-Biliary Surgery and Liver transplantation Unit, University of Modena, Italy.

**Fig. 4:**
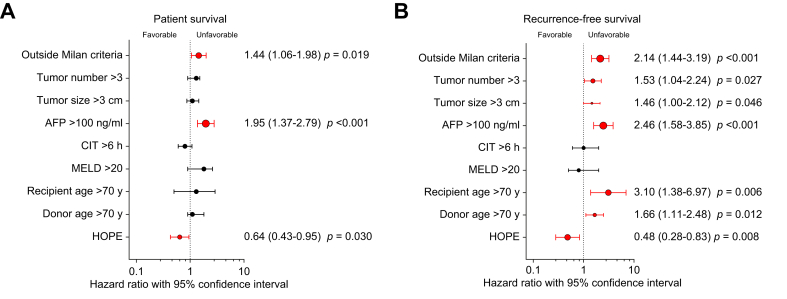
Multivariable Cox regression identifying independent predictors of survival in HCC liver transplant recipients. (A,B) Multivariable Cox regression analysis in 1,083 liver transplant recipients with HCC (599 from the HOPE-REAL cohort and 484 from the non-perfused control group), assessing overall patient survival (A) and recurrence-free survival (B). Statistically significant independent predictors are highlighted in red. AFP, alpha-fetoprotein; HCC, hepatocellular carcinoma; HOPE, hypothermic oxygenated machine perfusion; MELD, model for end-stage liver disease.

Finally, to investigate independent factors associated with HCC recurrence, a multivariable Cox regression analysis was performed on the total cohort of HOPE-treated (N = 599) and non-perfused (n = 484) HCC LT recipients. This analysis confirmed a favorable effect of HOPE on both recurrence-free and overall survival (Fig. 4). The specific HOPE modality (NRP-HOPE, DHOPE, or HOPE) had no significant impact.

## Discussion

This study represents the largest cohort of machine-perfused livers transplanted for HCC to date and demonstrates that HOPE-treated livers are associated with low HCC recurrence rates after DBD (7.1%) or DCD (6.4%) LT. These low recurrence rates correspond to excellent 5-year overall (81%) and recurrence-free (78%) survival, comparable to outcomes in non-HCC patients.

Machine perfusion of donor livers prior to transplantation has gained substantial attention over the past decade due to its ability to optimize grafts compared to conventional static cold storage,^12^ as well as its potential to evaluate graft viability before transplantation.^13^ Currently, multiple liver perfusion strategies are used in clinical practice: *in situ* NRP, *ex situ* normothermic machine perfusion (NMP), *ex situ* HOPE, or a combined, sequential approach. Each technique has distinct advantages and limitations.

HOPE is typically applied end-ischemically at the recipient center, after rapid procurement and static cold storage. HOPE reoxygenates ischemic tissue under hypothermic conditions, which decreases the risk of mitochondrial complex I dysfunction compared to normothermia,^14^ thereby reducing mitochondrial oxidative stress. During HOPE, ongoing NADH and succinate metabolism enables ATP synthesis,^15^ resulting in a metabolically resuscitated liver with high ATP and low succinate levels before implantation.^16^ This state contributes to reduced inflammatory signaling during reperfusion. It remains to be established whether application of HOPE already starting at the procurement center could yield even greater benefit.

*Ex situ* NMP can be applied either immediately after procurement, replacing cold storage, or end-ischemically at the recipient center, similar to HOPE.^17^ However, when NMP is used following a period of cold storage, the resulting warm reperfusion may trigger greater oxidative stress and inflammation compared to HOPE.^18^ In contrast, NRP is applied *in situ* in DCD donors offering tissue reoxygenation without the need for additional cooling. A key drawback of NRP, however, is the subsequent cold storage phase, which can lead to metabolic depletion before implantation.^19^ Based on these limitations, combined sequential strategies of NRP and HOPE,^20^ NRP plus NMP, or HOPE and NMP,^21^ have been introduced clinically in Italy and the Netherlands, particularly for DCD livers.

Tumor recurrence after liver surgery or transplantation is postulated to result from mobilization of circulating tumor cells, reactivation of dormant micro-metastases, or both.^5^ Surgical stress and IRI alter both the hepatic microenvironment and systemic immune landscape, potentially facilitating engraftment of tumor cells.^5^ The underlying mechanism is related to downstream consequences of ischemia reperfusion, *e.g*. the release of damage-associated molecular patterns, cytokines, adhesion molecules, and inflammasome activation.^5^ Accordingly, hepatic IRI can be divided into an acute phase, lasting approximately 3 h after reperfusion, and a late phase lasting up to 48 h after reperfusion. The acute phase is characterized by mitochondrial dysfunction causing oxidative stress in hepatocytes, Kupffer cells, and sinusoidal epithelial cells.^22^ Oxidative stress induces the release of HMGB1 (high-mobility group box 1), a damage-associated molecular pattern that mediates Kupffer cell activation through a toll like receptor 4-mediated pathway. Activated Kupffer cells produce more reactive oxygen species, which induce the degradation of IKB, a molecule that inhibits the translocation of NF-κB from the cytosol to the cell nucleus.^23^ Once freed, NF-κB enters the nucleus and upregulates the synthesis of mediators of the pro-inflammatory cytokine cascade, including IL-1b, IL-6, and TNF-a. Pro-inflammatory cytokines act on adhesion molecules and complement proteins to attract neutrophils and CD4+ T cells into the liver, which are the main mediators of the late phase of IRI. Neutrophils increase liver damage through secretion of reactive oxygen species and proteases causing hepatocyte necrosis.^24^ This liver damage can eventually evolve into chronic inflammation, causing long-term consequences such as liver fibrosis but also tumor growth by creating a favorable microenvironment for tumor cell engraftment or proliferation of dormant micro-metastases. Correspondingly, increased cold or warm ischemia times,[25], [26], [27] as well as donor-related risk factors,^3^ have been associated with higher HCC recurrence rates after LT. Machine perfusion, by mitigating IRI and dampening inflammation, may therefore offer a novel and clinically relevant approach to reduce tumor seeding and HCC recurrence.^4^^,^^5^

To date, however, data on the effects of NRP and NMP on HCC recurrence after LT remain scarce. Clinical evidence on HOPE in terms of cancer is also limited and somewhat conflicting. Mueller *et al.* reported lower HCC recurrence rates following DCD LT with HOPE,^8^ whereas Rigo *et al.* observed no difference.^28^ HOPE may also exert immunomodulatory effects, reducing rejection and allograft immunogenicity by suppressing direct alloantigen presentation.^29^ After HOPE treatment, LT recipients have demonstrated increased circulating regulatory T cells (CD4^+^CD25^+^FoxP3^+^), as well as a selective decrease in donor-specific CD8^+^ T cells 3 months after LT.^30^ Recent data on ischemia-free liver transplantation, a special form of NMP that entirely avoids ischemia during procurement, preservation, and implantation, showed significantly lower HCC recurrence compared to conventional LT.^31^ These findings further support the hypothesis that ischemia in fact plays a central role in post-LT HCC recurrence, and that either avoiding ischemia or mitigating IRI may reduce the risk of HCC recurrence after LT.

**Fig. 5:**
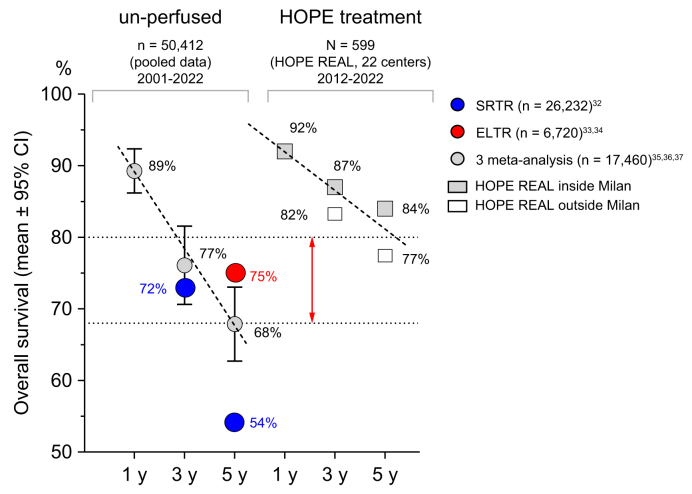
Comparison of 1-, 3-, and 5-year survival after HCC transplantation between HOPE-treated and historical non-perfused cohorts. One-, 3-, and 5-year survival rates for patients who underwent liver transplantation for HCC, comparing pooled data from previously published studies which exclusively included non-perfused livers (including US cohorts, the ELTR cohort, and three meta-analyses) with outcomes from the HOPE-REAL HCC cohort. ELTR, European Liver Transplant Registry; HCC, hepatocellular carcinoma; HOPE, hypothermic oxygenated machine perfusion; SRTR, Scientific Registry of Transplant Recipients.

Our findings align with this hypothesis. We observed low HCC recurrence rates in a large international cohort of HOPE-treated LT recipients, despite a high proportion of DCD grafts and a median donor age of 63 years. Notably, overall survival was comparable between propensity score-matched HCC and non-HCC recipients within the HOPE-REAL study. In an external propensity-matched comparison with non-perfused DBD livers, HOPE-treated HCC DBD recipients demonstrated significantly superior 5-year overall survival (Fig. 3). These outcomes compare favorably with previously published data from several large US**^32^** and European LT cohorts,[33], [34], [35], [36], [37] which report HCC recurrence rates of 15-19% and 5-year overall survival rates ranging from 54% to 76% in over 50,000 non-perfused HCC recipients (Fig. 5).

This study has several limitations. The median follow-up time in the HOPE-REAL study was 2.4 years; however, most HCC recurrences typically occur within this time frame. Additionally, the cohort included a relatively high proportion of low-risk HCC cases. Nevertheless, propensity score-matching ensured that the tumor burden in the HOPE-treated cohort was comparable to that of the external non-perfused control group. Third, the HOPE REAL cohort consisted of a much higher number of centers, while the control group included only two centers. However, we observed no center effect on HCC recurrence in the HOPE treated cohort and would therefore not consider the difference in participating centers as systematic bias. Another shortcoming is the lack of reporting on waiting times and potential bridging therapies in this observational study, as these data were not available retrospectively from all centers. In addition, non-standardized surveillance regimens could potentially have influenced the time to reported recurrence. These are all important shortcomings, which can likely only be well addressed by prospective multicenter trials, such as the ongoing HOPE4Cancer trial (NCT06717919), including more than 35 LT centers across Europe and the US, with standardized imaging for all patients and adjustment for pre- and post-transplant treatments as well as for tumor biology.^38^ This study may also provide an opportunity to investigate potential biomarkers of tumor recurrence.

In summary, this study demonstrates low recurrence rates and excellent long-term survival after LT for HCC using HOPE-treated grafts, with outcomes comparable to non-HCC recipients and superior to those receiving non-perfused livers. These results support the potential anti-tumor benefits of machine perfusion and warrant further validation in the ongoing multicenter HOPE4Cancer trial. As an *ex situ* treatment that is relatively straightforward, HOPE and potentially other perfusion strategies may have significant clinical implications in the evolving field of transplant oncology.

## Abbreviations

AFP, alpha-fetoprotein; DBD, donation after brain death; DCD, donation after circulatory death; DHOPE, dual hypothermic oxygenated machine perfusion; DAMP, damage-associated molecular pattern; HCC, hepatocellular carcinoma; HOPE, hypothermic oxygenated machine perfusion; IRI, ischemia-reperfusion injury; LDLT, living donor liver transplantation; LT, liver transplant(ation); MELD, model for end-stage liver disease; NMP, normothermic machine perfusion; NRP, normothermic regional perfusion; NRP-HOPE, normothermic regional perfusion followed by HOPE.

## Authors’ contributions

Study design: JE, PD, VdM. Funding acquisition: PD. Data collection: all co-authors. Data analysis: JE, PD, CK. Manuscript writing: JE, PD, VdM. Structured discussion: JE, PD, VdM. Manuscript revision: JE, PD, Vdm, VM.

## Data availability

The patient data that support the findings of this study are not openly available to legal and ethical restrictions associated with patient confidentiality. However, anonymized data can be made available from the corresponding authors upon reasonable request.

## Ethical committee approval

The medical Research Ethics Committee of the University Medical Center Groningen reviewed the study and waived the need for informed consent.

## Financial support

The research on Hypothermic Oxygenated Perfusion (HOPE) against cancer is funded by the 10.13039/100000001Swiss National Science Foundation (SNF) grant No 33IC30_22162/1 to PD.

## Conflict of interest

The authors declare no conflicts of interest pertaining to this manuscript.

Please refer to the accompanying ICMJE disclosure forms for further details.
